# Enhancing bovine immune, antioxidant and anti-inflammatory responses with vitamins, rumen-protected amino acids, and trace minerals to prevent periparturient mastitis

**DOI:** 10.3389/fimmu.2023.1290044

**Published:** 2024-01-08

**Authors:** Muhammad Zahoor Khan, Bingjian Huang, Xiyan Kou, Yinghui Chen, Huili Liang, Qudrat Ullah, Ibrar Muhammad Khan, Adnan Khan, Wenqiong Chai, Changfa Wang

**Affiliations:** ^1^ Liaocheng Research Institute of Donkey High-efficiency Breeding and Ecological Feeding, Liaocheng University, Liaocheng, China; ^2^ College of Life Sciences, Liaocheng University, Liaocheng, China; ^3^ Faculty of Veterinary and Animal Sciences, University of Agriculture, Dera Ismail Khan, Pakistan; ^4^ College of Life Science, Anhui Agricultural University, Hefei, Anhui, China; ^5^ Genome Analysis Laboratory of the Ministry of Agriculture, Agricultural Genomics Institute at Shenzhen, Chinese Academy of Agricultural Sciences, Shenzhen, China

**Keywords:** periparturient period, mastitis, dairy cattle, immunity, antioxidant status, antiinflammation, amino acids, trace minerals

## Abstract

Mastitis, the inflammatory condition of mammary glands, has been closely associated with immune suppression and imbalances between antioxidants and free radicals in cattle. During the periparturient period, dairy cows experience negative energy balance (NEB) due to metabolic stress, leading to elevated oxidative stress and compromised immunity. The resulting abnormal regulation of reactive oxygen species (ROS) and reactive nitrogen species (RNS), along with increased non-esterified fatty acids (NEFA) and β-hydroxybutyric acid (BHBA) are the key factors associated with suppressed immunity thereby increases susceptibility of dairy cattle to infections, including mastitis. Metabolic diseases such as ketosis and hypocalcemia indirectly contribute to mastitis vulnerability, exacerbated by compromised immune function and exposure to physical injuries. Oxidative stress, arising from disrupted balance between ROS generation and antioxidant availability during pregnancy and calving, further contributes to mastitis susceptibility. Metabolic stress, marked by excessive lipid mobilization, exacerbates immune depression and oxidative stress. These factors collectively compromise animal health, productive efficiency, and udder health during periparturient phases. Numerous studies have investigated nutrition-based strategies to counter these challenges. Specifically, amino acids, trace minerals, and vitamins have emerged as crucial contributors to udder health. This review comprehensively examines their roles in promoting udder health during the periparturient phase. Trace minerals like copper, selenium, and calcium, as well as vitamins; have demonstrated significant impacts on immune regulation and antioxidant defense. Vitamin B12 and vitamin E have shown promise in improving metabolic function and reducing oxidative stress followed by enhanced immunity. Additionally, amino acids play a pivotal role in maintaining cellular oxidative balance through their involvement in vital biosynthesis pathways. In conclusion, addressing periparturient mastitis requires a holistic understanding of the interplay between metabolic stress, immune regulation, and oxidative balance. The supplementation of essential amino acids, trace minerals, and vitamins emerges as a promising avenue to enhance udder health and overall productivity during this critical phase. This comprehensive review underscores the potential of nutritional interventions in mitigating periparturient bovine mastitis and lays the foundation for future research in this domain.

## 1 Introduction

Mastitis, characterized by the inflammation of mammary glands (1, 2), is intricately associated with immune suppression and an imbalance between free radicals and antioxidants within the animal’s physiological framework (3–5). Dairy cows confront the challenge of negative energy balance (NEB) during the periparturient period, precipitating a cascade of detrimental effects such as reduced dry intake, metabolic stress, hormonal fluctuations, heightened oxidative stress, and compromised immune responses (6, 7).

This NEB triggers the mobilization of fat reserves, culminating in the dysregulation of reactive oxygen species (ROS) and reactive nitrogen species (RNS) (8), along with elevated levels of non-esterified fatty acids (NEFA) (9, 10) and β-hydroxybutyric acid (BHBA) (11). The notable concentrations of NEFA and BHB compromise the bovine immune system, rendering dairy cattle more susceptible to infections (12). Scientific investigations have demonstrated that heightened NEFA and BHBA levels exert inhibitory effects on bovine peripheral blood mononuclear cells (BPMCs) (13), stifle interferon production (14), and impede the functionality of polymorphonuclear neutrophils (PMNLs) (15). Furthermore, escalated levels of NEFA and BHB serve as indicators of mastitis susceptibility (16, 17). During the periparturient period, the heightened concentrations of NEFA and BHB emerge as pivotal factors undermining immune function, directly augmenting the vulnerability to mastitis (11).

In addition to NEB-related factors, other metabolic disorders like ketosis and hypocalcemia indirectly contribute to mastitis in dairy cattle (18–20). Hypocalcemia prompts cows to spend more time lying down, resulting in teat exposure to physical injury and facilitating pathogen entry. The compromised sphincter muscle integrity of teats and diminished immune functionality due to reduced calcium levels emerge as pivotal factors linking mastitis with hypocalcemia.

In the realm of normal physiological conditions, the intricate antioxidant system adeptly mitigates and eradicates ROS stemming from metabolic processes. Nevertheless, the transitions accompanying pregnancy and calving instigate an overabundance of ROS production (21, 22). This disruption in the equilibrium between ROS generation and the availability of antioxidants ushers in oxidative stress, rendering cattle more susceptible to a spectrum of maladies (23, 24). The unrestrained ROS production culminates in lipid peroxidation, tissue impairment, and fluctuations in reduced glutathione (GSH) levels, a pivotal constituent of glutathione metabolism (24, 25). Oxidative stress inflicts damage upon the structure and function of cellular macromolecules, including lipids, proteins, and nucleic acids, thereby inciting metabolic dysfunctions and ailments, notably mastitis in dairy cattle (6, 26). Sustaining redox homeostasis during the periparturient and peak lactation phases is of paramount importance (27–29). Oxidative stress associated with parturition may contribute to immunological and inflammatory aberrations, heightening the vulnerability to metabolic and infectious disorders (22, 30).

The metabolic stress encountered during the periparturient period stands as another pivotal factor exposing animals to immune suppression and the deviant regulation of oxidative stress. This phase instigates excessive lipid mobilization, subsequently leading to oxidative stress (16). The abnormal regulation of immunity and inflammation driven by metabolic and oxidative stress constitutes the third critical factor predisposing dairy cattle to periparturient mastitis (3, 31–33). Furthermore, oxidative and metabolic stress, negative energy balance, immune suppression, and diminished productive efficiency collectively undermine the overall productivity of these animals (34–36).

Numerous nutritional strategies have been employed to address the challenges encountered during the periparturient period in dairy cattle (37–39). Extensive research endeavors have been undertaken to probe the intricate interplay between nutrition, immune modulation, and the augmentation of antioxidant status, thereby fostering enhanced udder health during this critical phase (40–45). O’Rourke (41), in particular, highlighted that cows undergoing negative energy balance face an elevated risk of ketosis, with clinical ketosis being associated with a twofold increase in the likelihood of clinical mastitis. Furthermore, extant research underscores the pivotal role of nutritional disturbances during the periparturient period in contributing significantly to immune suppression, oxidative stress, and metabolic perturbations (46–48).

Among the pivotal nutrients that have garnered attention, specific amino acids, trace minerals, and vitamins have exhibited a pronounced impact on udder health during the periparturient period, particularly in the prevention of mastitis in dairy cattle. Consequently, this review aims to provide a comprehensive exploration of the contributions made by trace minerals, vitamins, and amino acids in bolstering udder health during the critical periparturient phase in dairy cattle.

## 2 Key factors associated with periparturient bovine mastitis

### 2.1 Metabolic stress, NEFA and BHBA

The NEFA and BHBA represent metabolites resulting from the mobilization of fat reserves triggered by NEB during the perinatal period in dairy cows, exerting detrimental effects on the cellular physiology of various bovine cell types (12). Severe NEB initiates lipid mobilization, leading to elevated circulating concentrations of NEFA and BHBA. Clinical data derived from previous studies have established a strong correlation between heightened levels of NEFA and BHBA and an increased incidence of postpartum diseases, including mastitis, ketosis, clinical endometritis, metritis, and other conditions associated with immunosuppression. These conditions have adverse repercussions on the overall health, longevity, as well as the productive and reproductive performances of dairy cows (11, 49).

Furthermore, a study conducted by Li et al. revealed that NEFA and BHBA lead to increased accumulation of malondialdehyde (MDA) and ROS, along with reduced total superoxide dismutase (T-SOD) and glutathione peroxidase (GSH-Px) activity, resulting in oxidative stress. Additionally, NEFA and BHBA stimulation led to heightened expression of inflammatory markers such as nitric oxide (NO), tumor necrosis factor-alpha (TNF-α), interleukin-6 (IL-6) and interleukin-1 beta (IL-1β). Mechanistically, their data demonstrated that NEFA and BHBA activate the mitogen-activated protein kinase (MAPK) signaling pathway, shedding light on the fact that NEFA and BHBA induce oxidative stress and an inflammatory response, potentially via the MAPK signaling pathway in BMECs (12). Similarly, another study observed that elevated NEFA levels increased ROS levels, thereby activating the MAPK signaling pathway and triggering ER stress-mediated apoptosis in BMECs (50).

### 2.2 Oxidative stress

**Figure 1:**
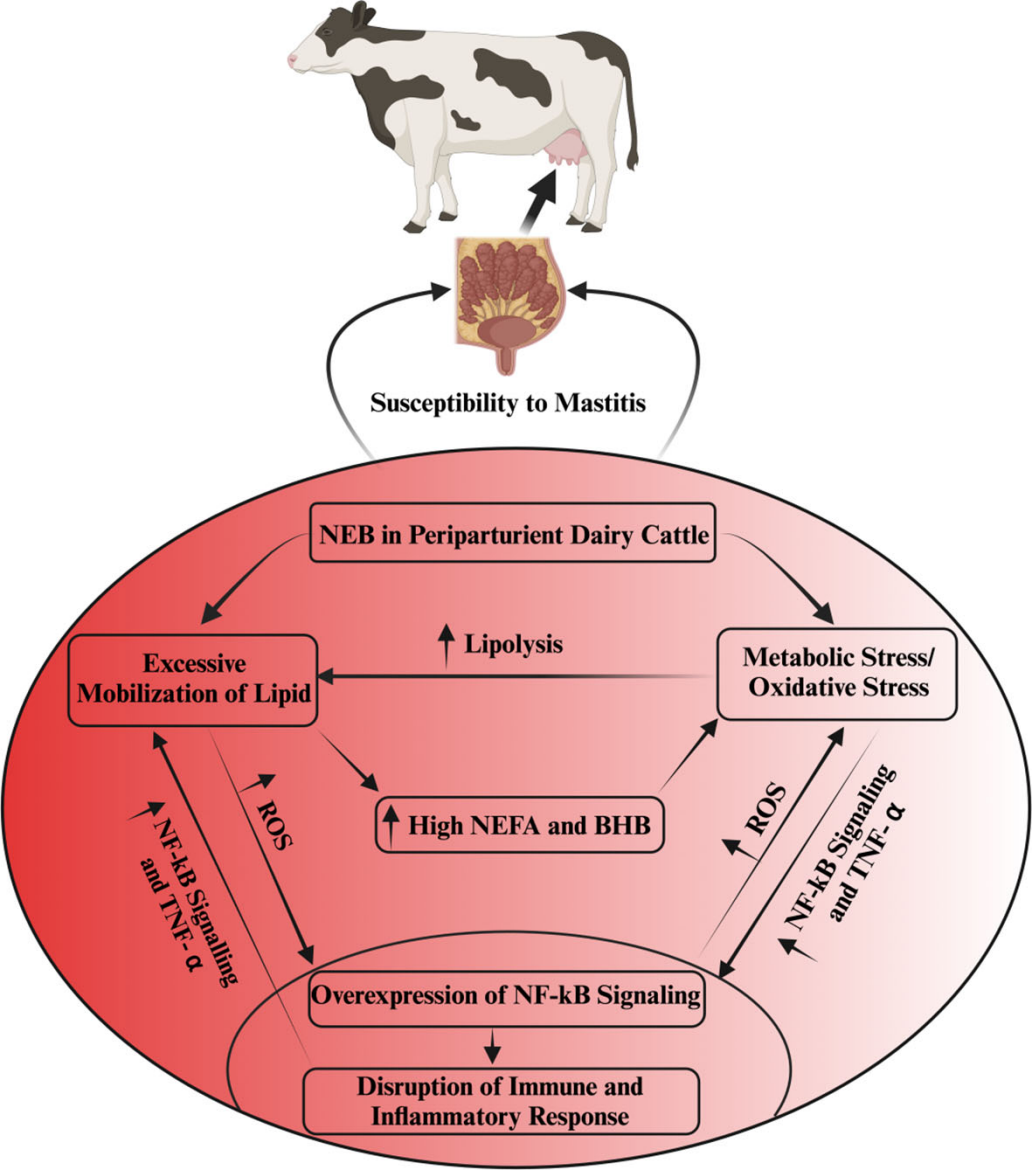
The interlink among metabolic stress, immunity, inflammation and mastitis susceptibility. Oxidative stress disrupts immune and inflammatory functions via the activation of NF-kB signaling. This aberrant inflammatory regulation, in turn, fosters excessive TNF-α production in non-phagocytic cells, resulting in heightened oxidative stress and increased lipolysis. The intricate interplay of oxidative stress, escalated lipid mobilization, and compromised immune and inflammatory processes is predominantly associated with NEB in periparturient dairy cattle. NEB, by triggering an excessive lipid mobilization in these cattle, elevates NEFAs, BHB and ROS, consequently inducing oxidative stress. This oxidative stress further exacerbates the disruption of immunity and inflammation regulation, rendering dairy cattle more susceptible to mastitis.

Oxidative stress induced by metabolic stress is closely associated with several pathological conditions, including mastitis, during the periparturient period in dairy cattle (26, 51, 52). Consistently, Sordillo and Aitken (3) reported that oxidative stress resulting from negative energy balance is intricately linked with impaired immunity, subsequently leading to heightened inflammation, thereby rendering dairy cattle more susceptible to mastitis (3, 53; 54). In a similar vein, a study noted that selenium supplementation effectively enhanced the total antioxidant capacity of dairy cattle, consequently mitigating the risk of mastitis in periparturient dairy cattle (55). Additionally, when BMECs were exposed to lipopolysaccharide (LPS), it was observed that ROS levels increased, accompanied by inflammatory changes (56, 57). In **Table 1**, we present a comprehensive summary of recent published research findings that elucidate the intricate relationship between oxidative stress and mastitis in dairy cattle during the critical periparturient period. The interplay among multiple factors, including metabolic stress/oxidative stress, immunity, inflammation, NEB, and susceptibility to mastitis, has been depicted and summarized in **Figure 1**.

**Table 1:** Recent studies reported association of metabolic disturbances, suppressed immunity and oxidative stress with periparturient bovine mastitis.

S.No	Outcomes of oxidative stress	Impact on udder health of dairy cattle	References
1	Impaired immune response and abnormal regulation of inflammation by metabolic induced oxidative stress	Susceptible to mastitis	5, 36, 56, 58
2	Inhibition of nuclear factor erythroid 2 related factor 2 (NFE2L2), a master regulator of cellular redox homeostasis was followed by decreased level of GSH-Px, catalase (CAT), and superoxide dismutase (SOD) and increased level of ROS and MDA in response to exogenous free fatty acids (FFA) in bovine mammary epithelial cells	Induced inflammatory changes in mammary gland	59
3	Increased levels of ROS, oxidative stress index (OSi), and decreased *α*-tocopherol (*α*-T) and serum antioxidant capacity (SAC) levels around parturition	At high risk of developing mastitis	60
4	The NEFA and BHBA induced oxidative stress and suppressed immunity in BMECs	Increased chances of mastitis	12, 50
5	Low level of GSH, SOD, CAT, and total antioxidant capacity (T-AOC); higher levels of ROS and MDA	Associated with mammary gland inflammation	61
5	Elevated oxidative stress	Increased the susceptibility of dairy cattle to mastitis	62
6	The progressive development of oxidative stress during the transition from late gestation to peak lactation is thought to be a significant underlying factor leading to dysfunctional immune cell responses.	Expose dairy cattle susceptibility to infections including mastitis	55
7	Elevated level of serum amyloid A (SAA), MDA and decreased level of total antioxidant capacity	Increased risk of mastitis	63

## 3 Trace minerals role in bovine mastitis prevention during periparturient period

### 3.1 Reference values for serum trace minerals in dairy cows

To establish appropriate dietary recommendations, it is essential to consider reference values for serum trace mineral concentrations in dairy cows. Notable values include: calcium (2.2–2.6 mmol/L) (64), phosphorus (1.3–2.6 mmol/L) (64), magnesium (0.75–1.0 mmol/L) (65), selenium (0.73–1.08 µmol/L) (66), copper (1–18 µmol/L) (67), and zinc (8–19 µmol/L) (68). Meeting these reference values can help optimize the mineral supply and overall health of dairy cattle.

### 3.2 Role of trace minerals in health regulations of dairy cattle

In the field of bovine veterinary medicine, the pivotal role of mineral deficiencies in modulating the immune system should not be underestimated. Recent investigations have elucidated that the provision of micronutrient supplements has demonstrably reduced the count of milk somatic cells (SCC), bolstered immune function, and elicited anti-inflammatory and antioxidant responses in dairy cattle during the periparturient period (69, 70). Extensive research has shown that these deficiencies can lead to immunosuppression, making the animals more susceptible to infectious diseases such as mastitis (42). Consequently, addressing mineral imbalances in cattle health becomes crucial in maintaining optimal health outcomes. Minerals typically constitute essential structural components within the body, acting as crucial cofactors for diverse enzymes and participating in vital processes such as nerve signaling, muscle contraction, and the regulation of proper keratosis. Insufficient mineral levels can result in diminished immune cell activity or disruption of innate defense mechanisms within the breast, thereby fostering the progression of mastitis (71). Recent studies have shown promising results regarding the supplementation of trace minerals and its effect on dairy cattle health by enhancing their immune and antioxidant status during the periparturient period (72–74). Trace mineral supplementation has been found to significantly enhance immune and antioxidant status, contributing to reduced levels of NEFA (75) and alleviating inflammatory changes, which are critical factors associated with clinical mastitis (76–80). These studies emphasize the potential of mineral supplementation as a strategy to improve cattle health and overall herd performance. Minerals play a fundamental role in maintaining the immune system of cattle. Recent studies have highlighted the association between serum trace mineral concentrations and their impact on bovine health. For instance, higher concentrations of serum selenium and phosphorus have been linked to the successful cure of bovine clinical mastitis (81). Similarly, research demonstrated that subcutaneous supplementation of specific minerals, such as zinc, manganese, selenium, and copper, led to increased SOD activity, decreased serum BHBA concentrations, reduced milk SCC, and a lower incidence of mastitis (82). These findings underline the vital role of trace minerals in preventing and mitigating the effects of mastitis in dairy cattle.

In clinical practice, certain mastitis cases may require supportive therapy, including the administration of calcium-containing fluids, due to the prevalent occurrence of hypocalcemia in cows with udder inflammation (83). Additionally, the supplementary use of injectable trace minerals, such as zinc, manganese, selenium, and copper, has been considered as an adjunctive tool in mastitis therapy. Clinical evidence suggests that supportive therapy involving the administration of fluids enriched with calcium can be crucial in managing mastitis in dairy cows (83). Hypocalcemia, commonly observed in cows with udder inflammation, can compromise immune function and hindering the healing process. Proper calcium supplementation has shown promise in improving the overall therapeutic outcomes in mastitis cases. Recent studies have investigated the use of injectable trace minerals as a complementary approach in the treatment of mastitis in dairy cows. Hoque et al. (84) emphasized the significance of antimicrobial therapy as the primary treatment for mastitis. However, their experiment revealed a noteworthy finding – cows that received only selenium preparations demonstrated reduced susceptibility to udder inflammation compared to the untreated control group. Ganda et al. (81) conducted a study involving the injection of trace minerals, including zinc, manganese, selenium, and copper, in dairy cows with mastitis. The results indicated a reduction in the number of chronic mastitis cases, showcasing the potential of injectable trace minerals in mitigating the severity and persistence of the disease.

**Figure 2:**
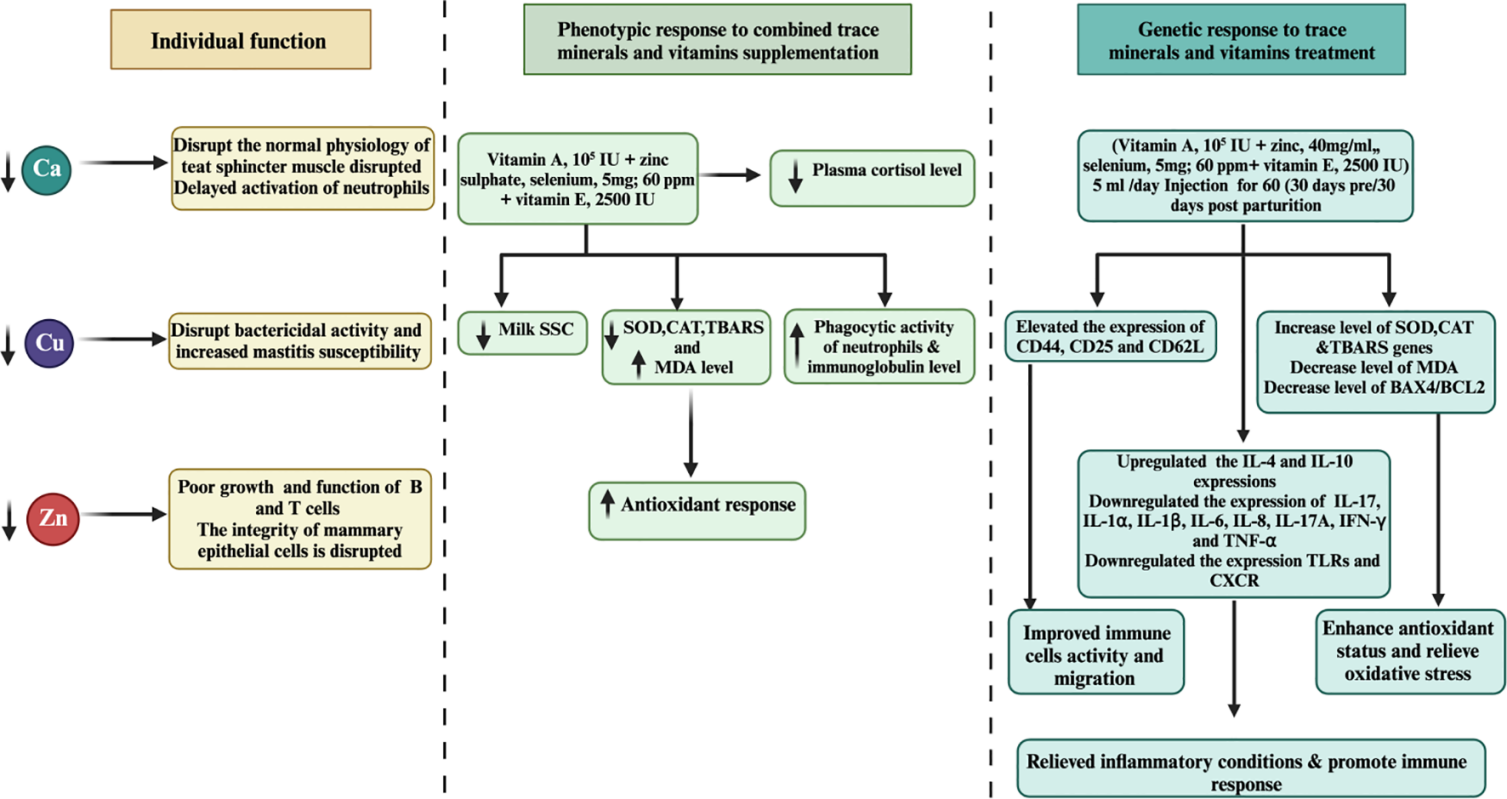
Associations between molecular and phenotypic factors related to immunity, apoptosis, milk SCC, anti-inflammatory responses, and antioxidant activity in dairy cattle following combined treatments with minerals and vitamins are examined. Over a 60-day duration, the combined treatment of minerals and vitamins exhibited notable downregulation of apoptosis and inflammation-related gene expression levels, concomitant with upregulation of genes associated with immunity, antioxidation, and pro-inflammatory responses in periparturient dairy cattle. Furthermore, the integrated regimen of trace minerals and vitamins demonstrated enhancements in neutrophil phagocytic activity, accompanied by reductions in plasma cortisol and milk SCC levels, suggesting a potential association with mastitis prevention during the periparturient period in dairy cattle.

Another noteworthy study by Machado et al. (85) explored the effects of injecting a multimineral preparation containing selenium, copper, zinc, and manganese. Their findings demonstrated a positive impact on udder health, leading to a decrease in linear SCC, and a reduction in the incidence of subclinical and clinical mastitis cases. Furthermore, the researchers reported an increase in serum SOD activity, indicating potential antioxidant benefits without affecting leukocyte function (82). Despite the positive effects observed in other studies, Ferreira and Petzer (86) reported no significant correlation between SCC and the levels of selenium in milk or serum among cows supplemented with selenium in various forms. This suggests that other factors might influence the relationship between SCC and selenium supplementation. A separate investigation by Bourne et al. (87) investigated the supplementation of vitamin E with selenium. The study revealed a notable 10% reduction in the risk of culling and mastitis rate, suggesting a potential synergistic effect between vitamin E and selenium in enhancing mastitis treatment outcomes. Recent research conducted by Smulski et al. (88) indicated that combining an antibiotic treatment with an antioxidant containing selenium slightly improves the overall effectiveness of clinical mastitis treatment. This highlights the importance of considering combination therapies to optimize mastitis management in dairy cows. Further investigation is necessary to gain a comprehensive understanding of its effects on cattle health and immune responses. **Figure 2** illustrates the pivotal role of trace mineral supplementation in health regulation, encompassing its capacity to enhance immune function, mitigate inflammation, and bolster antioxidant responses among periparturient dairy cattle.

### 3.3 Specific

#### 3.3.1 Role of calcium, copper, zinc, and selenium in regulating anti-inflammatory and immune responses to prevent mastitis

Calcium assumes a pivotal physiological role within the biology of living organisms, functioning as an indispensable constituent of bodily structures and a critical determinant for muscle contractions, encompassing both skeletal and smooth muscles, such as the mammary gland sphincter. A prominent periparturient metabolic affliction, known as hypocalcemia, arises due to the excessive depletion of calcium in colostrum and milk. Consequently, the requisite calcium level is constrained to remain below 1.5 mmol/L to maintain normal muscle physiology, resulting in afflicted animals assuming a protracted recumbent posture (89). Furthermore, diminished calcium levels also correlate with compromised phagocytic activity of neutrophils and suppressed immunity, predisposing dairy cattle to other periparturient diseases (90, 91). In addition, the incapacity of the mammary gland sphincter muscle to contract leads to prolonged teat opening, creating favorable conditions for pathogenic microorganisms to induce mastitis (92). Notably, a study revealed lower calcium levels in acute *Escherichia coli (E.coli)* mastitic cows compared to healthy counterparts (93). Moreover, investigations have unveiled that certain polymorphisms (G519663A and G38819398A) within the Calcium channel, voltage-dependent, alpha-2/delta subunit 1 (CACNA2D1) gene are associated with mastitis resistance in Sahiwal cattle (94, 95).

Copper is another trace mineral, plays a crucial role in the structure and catalytic properties of cuproenzymes, which are enzymes that incorporate copper (96). Notable examples of cuproenzymes include cytochrome-c oxidase, superoxide dismutase, catechol oxidase, ceruloplasmin, and amine oxidases. Additionally, copper holds a significant position among enzymes, ranking as the second most prevalent metal after zinc and contributing to their proper functioning (97). Apart from its involvement in enzymatic functions, copper also plays a vital role in various physiological processes. It contributes to essential processes such as collagen and elastin synthesis, which are crucial for tissue structure and elasticity. Furthermore, copper is involved in myelination, a process critical for nerve impulse conduction, and hemoglobin production, which is essential for oxygen transport (96). Copper has been recognized for its antibacterial properties against bacteria commonly isolated from mastitic cows. Research by Reyes-Jara et al. (98) highlights that even low copper concentrations, as low as 250 ppm, can effectively inhibit the growth of mastitis microorganisms like *E.coli* and coagulase-negative *Staphylococci*. Supporting studies by Wernicki et al. (99) and Kalińska et al. (100) demonstrate the potent antimicrobial activity of silver and copper nanoparticles against bacteria derived from inflamed udders, indicating copper preparations as potential alternatives to dipping solutions. *In vivo* investigations have revealed promising results concerning the effects of copper supplementation. A 100-day dietary copper supplementation study, as conducted by Scaletti et al. (101), showed a reduced clinical response in Holstein cows experimentally intramammary infected with *E. coli*. The experimental group, supplemented with copper at a concentration of 20 ppm, exhibited improved outcomes compared to the control group with 6.5 ppm copper concentration. Likewise, Gakhar et al. (102) observed a decreased incidence of postpartum mastitis in cows supplemented with copper, further validating the potential benefits of copper supplementation. Copper deficiencies have been associated with impaired phagocytosis and decreased Cu, Zn-SOD (copper-zinc superoxide dismutase) activity. These findings underscore the importance of adequate copper levels for a properly functioning immune system (103). The antibacterial properties of copper are explained by the oxidation-mediated disruption of bacterial lipids, proteins, and DNA. This mechanism highlights the potential of copper as an effective antimicrobial agent (104).

Zinc is a vital trace element that plays a pivotal role in the maintenance of rumen microbiota and the synthesis of essential proteins, including collagen, glucagon, insulin, DNA, and RNA (105). It serves as an indispensable activator for a diverse array of enzymes, encompassing alkaline phosphatase, carbonic anhydrase, DNA and RNA polymerase, and, in conjunction with copper, superoxide dismutase, which assumes a key role in antioxidant processes (97). Furthermore, zinc acts as a crucial co-factor for a series of oxidoreductases and significantly contributes to keratin formation. Studies have shown that dietary supplementation of zinc can have a significant impact on the health of dairy cattle. Specifically, some research has reported that zinc supplementation results in reduced somatic cell count (106, 107) and decreased milk amyloid A levels (106). However, contrasting findings were observed by Whitaker et al. (108), where no effect of dietary zinc supplementation on SCC was found. The integrity of the intact mammary epithelium, which acts as an impermeable barrier to microorganisms, is recognized as an intrinsic component of the udder immune system. Notably, studies have demonstrated that the supplementation of zinc preparations in Holstein cows can lead to improved integrity of the mammary epithelium (109), although contradictory results have been reported by Shaffer et al. (110). Zinc is crucial for the development and proper functioning of cells involved in innate immunity, such as neutrophils. Deficiency of this essential mineral adversely affects the growth and function of T and B cells, impacting the overall immune response in dairy cattle. Zinc exhibits potent antioxidant properties and plays a pivotal role in stabilizing cellular membranes. This feature suggests its significance in preventing free-radical-induced injuries during inflammatory processes (111).While the supplementation of trace minerals has demonstrated the capacity to augment immune responses and ameliorate inflammatory alterations, it is imperative to emphasize the need for extensive and in-depth investigations aimed at elucidating the precise underlying mechanisms and exploring the potential therapeutic applications of such supplementation in the context of mitigating mastitis in dairy cattle.

#### 3.3.2 Role of selenium of in regulation of inflammatory and immune response to prevent mastitis

Selenium has garnered considerable attention as a crucial element with antioxidant and immune-regulatory properties (36). Extensive research, encompassing *in-vitro* and *in-vivo* studies, has demonstrated that Se supplementation can alleviate inflammatory changes, oxidative stress, and mastitis caused by *S. aureus* in mice mammary glands (112–114). Plasma GPx-3, an extracellular antioxidant protein containing selenocysteine, plays a vital role in reducing hydrogen peroxide and lipid hydroperoxides (115). GPx-3 is essential for the antioxidant defense mechanism in dairy cattle (116). Furthermore, milk lactoserum obtained from selenium-fed cows has shown undefined antibacterial action, potentially attributed to the elevated level of GSH-Px resulting from selenium treatment (117–119). Selenium supplementation has also been found to regulate antioxidant-associated genes (SOD, GPX, TOAX, GSH, and CAT) and suppress oxidative stress in dairy cattle, thus preventing oxidative stress and subsequent infections (120). The pivotal role of selenium in regulating immunity and antioxidant activity has made it a primary focus in mastitis control research for dairy cattle. Studies have indicated a positive correlation between low levels of selenium and GPx with oxidative stress in periparturient dairy cattle. However, desired selenium supplementation in mammary epithelial cells has been found to promote anti-oxidative responses, leading to a reduction in apoptotic cells (121). Moreover, low GPx levels in blood were associated with a higher percentage of mammary infections (mastitis) in periparturient dairy cows (122). Recent research indicates that S. aureus has the ability to modulate myeloid differentiation factor 88 (MYD88) and engage the NF-kB signaling pathway, thereby initiating inflammatory responses within the mammary gland through its interaction with toll-like receptor 2 (TLR-2) (123, 124). In a recent investigation by Wei et al. (125), an elevation in MYD88, IL-1β, TNF-α, pyrin domain-containing protein 3 (NLRP), caspase-recruitment domain (ASC), and caspase-1 levels was observed in *S. aureus-*infected macrophages in mice. Remarkably, a 90-day regimen of selenium treatment led to a significant reduction in the expression of MYD88, IL-6, IL-1β, NLRP3, and ASC within the macrophages (125).

During instances of microbial infection and cellular damage, the NLRP3 inflammasome—a vital component of the innate immune system—maintains regulation over caspase-1 activation and the secretion of pro-inflammatory cytokines such as IL-1β/IL-18. However, *S. aureus-*mediated irregular control of the NLRP3 inflammasome within the mammary gland leads to an atypical inflammatory response (126, 127). Supplementing with selenium was shown to significantly curtail the expression of NLRP3 inflammasome and proinflammatory cytokines IL-1β/IL-18, thereby mitigating the abnormal inflammatory response and consequently preventing mastitis in mice (112, 127). Furthermore, Se supplementation effectively restrained the nuclear transcription factor-kappa B (NF-κB) and MAPK signaling pathways that are intricately involved in the progression of mastitis within murine macrophages (128, 129). This suggests that selenium supplementation holds potential in suppressing the inflammatory alterations elicited by *S. aureus.*

NF-κB and MAPK signaling pathways wield pivotal roles in initiating inflammatory transformations by bolstering cytokine production during *S. aureus-*induced mastitis in the mammary gland (113, 130, 131). This observation is consistently upheld by Liu et al. (113), who found that LPS supplementation led to a reduction in the recruitment of neutrophils and macrophages in mammary epithelial cells. Moreover, the overexpression of TLR2, IL-1β, TNF-α, and IL-6, coupled with the heightened phosphorylation of NF-κB and MAPKs proteins resulting from *S. aureus* exposure, were markedly alleviated through Se supplementation in mouse models (92, 112, 113, 132).

**Figure 3:**
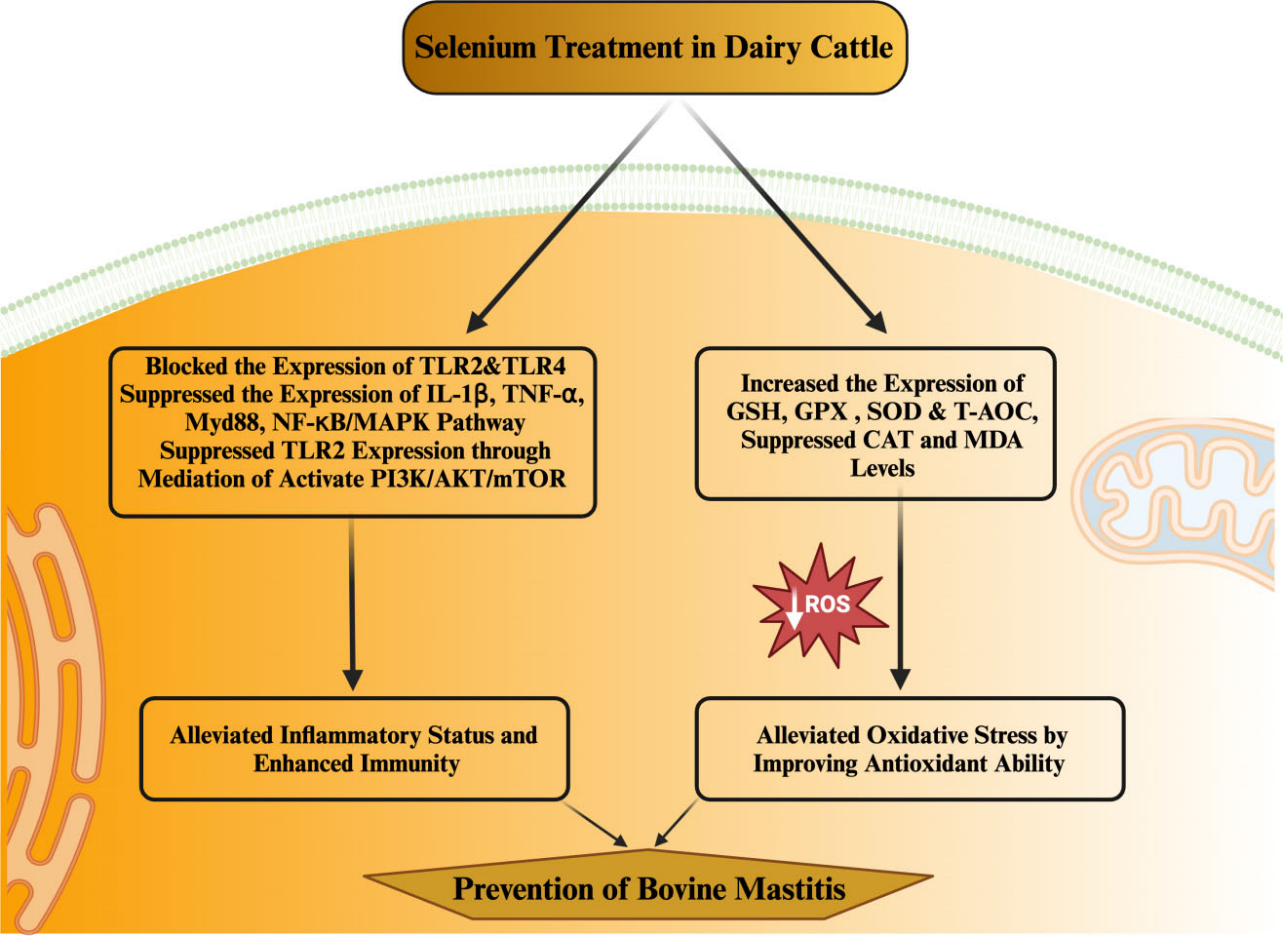
Mechanism through which selenium prevent mastitis in dairy cattle during periparturient period in dairy cattle.

MerTK has been extensively documented as a key player in regulating the PI3K/AKT/mTOR pathway to enhance anti-inflammatory capabilities. Activation of the PI3K/Akt pathway within macrophages by MerTK can effectively impede NF-κB signaling (133). Furthermore, MerTK-mediated modulation of the PI3K/AKT/mTOR pathway exhibited repressive effects on TLR2-triggered immune responses, resulting in diminished inflammatory reactions and oxidative stress in U937 cells (134). *S. aureus* in its induction of an inflammatory response within the mammary gland of mice increased the levels of IL-1β, IL-6, and TNF-α. Notably, treatment with S. aureus led to decreased phosphorylation levels of MerTK, PI3K, AKT, and mTOR in the mouse mammary gland (114). Conversely, selenium treatment enhanced the phosphorylation levels of MerTK, PI3K, AKT, and mTOR while concurrently reducing the expression of inflammatory cytokines (IL-1β, IL-6, and TNF-α). These observations underscore selenium’s potential to augment immunity, enhance antioxidant status, and mitigate the inflammatory response within mammary glands, thereby alleviating mastitis in mice (114). In a recent *in vitro* experimental trail utilizing mammary alveolar cell large T antigen (MAC-T), Jing et al. (135) substantiated that selenium treatment had a marked downregulatory effect on genes (IL1B, IRAK4, MYD88, and SOCS3) linked to mastitis progression in dairy cattle. Furthermore, selenium treatment exhibited inhibitory effects on PI3K/AKT, MAPK, and NF-κB signaling pathways, while concurrently promoting the anti-inflammatory milieu through acceleration of the PI3K/Akt/mTOR pathway in dairy cattle (135). Furthermore, documented evidence indicates that Se can enhance the expression of IL10 and peroxisome-proliferator-activated receptor gamma (PPAR-γ) activity, while concurrently suppressing NF-κB and NO within the mammary gland. These effects hold true for cases of *S. aureus-*induced mastitis (136, 137). Additionally, selenium has been shown to alleviate the oxidative stress and inflammatory state of the mammary gland—a crucial factor in reducing the susceptibility of mice to mastitis (136; Zhang W et al., 2016). Various investigations have demonstrated positive correlations between selenium intervention and notable outcomes, including diminished SCC in milk, elevated levels of GSH-Px and reduced concentrations of MDA in plasma (138–144). Higher MDA levels are directly associated with oxidative stress, which heightens the susceptibility of periparturient dairy cattle to mastitis. Within the realm of animals, a group of twenty-five selenoproteins, twelve of which exhibit antioxidant and immunological functions, have garnered attention as promising candidates for mitigating mastitis in transition dairy cattle (135, 145, 146). The selenium content appears to be a determining factor influencing the vulnerability of mammary glands to bacterial infections during the periparturient period in dairy cattle (122). Additionally, a notable observation was made regarding selenium treatment’s efficacy in reducing SCC levels and IL-6 concentrations, while concurrently augmenting GSH-Px activity in dairy cattle (122). The potential of selenium treatment in impeding the growth of mastitis-causing bacteria, such as *Streptococcus uberis (S. uberis), S. aureus, E. coli*, and *Streptococcus agalactiae*, has been documented (147). This approach has also been associated with pronounced antibacterial effects, heightened antioxidant capacity, elevated GSH-Px activity, and lowered SCC levels in the milk of perinatal dairy cows (147). Likewise, other investigations have reported that selenium intervention, by enhancing GSH-Px activity, reduces the likelihood of mammary gland infections in dairy cattle during the transition phase (148, 149). Similarly, a distinct finding was noted, where selenium supplementation outperformed antibiotic treatment during the periparturient period in dairy cattle. To elaborate, among 36 cows, 14 still encountered infections even after antibiotic treatment, while merely 4 out of 36 cows developed mastitis in response to 4 mg of selenium supplementation during the transition phase in dairy cattle (150). Additionally, records indicate that selenium intake enhanced antioxidant capacity and concurrently regulated both innate and adaptive immunity within the mammary glands, effectively countering mastitis in dairy cattle (117). The summary of the studies discussing the association of selenium with health-enhancing phenotypic traits (antioxidant, anti-inflammation, and immunity) has been presented in **Table 2**. In addition, the mechanism through which selenium prevents mastitis has been highlighted in **Figure 3**.

**Table 2:** Selenium role in alleviation of mastitis by improving immunity, anti-inflammatory and antioxidant status of dairy cattle.

S.No	Treatment	Biological impact	References
1	Selenium treatment (1.5 mg/kg) of *S. aureus* infected mammary gland cells (inoculation of 100 μl *S. aureus* with 7 × 10^8^ CFU/μl)	• Mastitis induced by *S. aureus* within the mammary gland tissue of mice was driven by the upregulation of specific elements, including interleukin (IL-1β), IL-6, TNF-α, NF-κB, and MAPK pathways. • Conversely, the introduction of selenium supplementation in mice acted as a preventive measure against *S. aureus-*induced mastitis. • This was achieved by effectively inhibiting the expression levels of IL-1β, IL-6, TNF-α, NF-κB, and MAPK pathways. • Furthermore, the application of selenium treatment in mice yielded additional benefits; it not only curtailed the inflammatory response but also alleviated oxidative stress resulting from injuries to the mammary gland tissues caused by *S. aureus.*	Liu et al. (113)
2	Organic selenium (20 μg/kg body weight/day) treatment of *S. aureus* infected mammary gland cells ((2 × 10^7^ CFU/mL)	• In the context of *S. aureus*, the initiation of inflammatory alterations occurred through the augmentation of inflammatory cytokines’ expression (IL-1β, IL-6, and TNF-α), coupled with the reduction in the phosphorylation levels of MerTK, PI3K, AKT, and mTOR. • Contrastingly, selenium treatment orchestrated a different outcome: it effectively lowered the levels of IL-1β, IL-6, and TNF-α expression. Simultaneously, it bolstered the phosphorylation levels of MerTK, PI3K, AKT, and mTOR. • This intricate signaling cascade facilitated an anti-inflammatory response, enhanced antioxidant status, and ultimately mitigated the onset of mastitis in the rat population.	Chen et al. (114)
3	Selenium (0.2 mg of Se/kg) treatment of *S. aureus (*(10^5^ CFU/mL) infected mammary gland cells	• In the context of *S. aureus* exposure, there was an observable increase in the expression of NLRP3, ASC, caspase-1, caspase-1 p20, and pro-IL-1β, thus intensifying the inflammatory response. • Conversely, the supplementation of selenium exerted a substantial inhibitory effect on the levels of NLRP3, ASC, caspase-1, caspase-1 p20, and pro-IL-1β. • This outcome stands as compelling evidence that selenium treatment serves as a preventive measure against *S. aureus-*induced mastitis in mice, achieved by effectively curtailing the NLRP3 level.	Bi et al. (127)
4	Selenium treatment (1.5 mg/kg) of *S. aureus* infected mammary gland cells (inoculation of 100 μl *S. aureus* with 7 × 10^8^ CFU/μl)	• The application of selenium treatment effectively curtailed the levels of NLRP3, IL-1β, TNF-α, ASC, and caspase-1 that resulted from *S. aureus* influence within the mammary gland of mice. Moreover, Se supplementation notably bolstered the antioxidant capacity, enhanced the anti-inflammatory response, and fortified the immune status of the mice population. • Beyond this, the administration of selenium emerged as a preventive strategy against mastitis, primarily attributed to its capability to inhibit NLRP3 inflammasome activation and suppress NF-κB/MAPK pathway signaling.	Ma et al. (112)
5	Selenium (2.0 μmol Se/L) treatment of *S. aureus* infected mammary gland cells	• Dietary supplementation with selenium led to a decrease in the expression of TLR2 and the activation of the NF-κB/MAPK pathway induced by *S. aureus* in murine subjects.	Wang L et al. (138)
6	Selenium (1.5/kg) treatment of *S. aureus* infected mammary gland cells	• *In vivo* and *in vitro* investigations demonstrated that *S. aureus* triggered inflammation, both within live organisms and primary mouse epithelial cells (MMECs) cultured in the laboratory. • This resulted in an increase in the expression levels of mmu-miR-155, IL-1β, TNF-α, and TLR2. Additionally, the phosphorylation levels within the NF-κB/MAPK signaling pathway were heightened in mammary epithelial cells of mice upon infection with *S. aureus.* • Remarkably, selenium displayed a notable capacity to suppress the elevated expression levels of mmu-miR-155, IL-1β, TNF-α, TLR2, and the NF-κB/MAPK signaling pathways within the mammary epithelial cells of mice. These insightful findings suggest that selenium holds potential in averting mastitis in mice by mitigating oxidative stress and curtailing the inflammatory response.	Zhang et al. (92)
7	Selenium (1.5 mg/kg) treatment of *S. aureus* infected mammary gland cells	• Selenium intervention mitigated both oxidative stress and the inflammatory reaction. It downregulated the expression of IL-1β, TNF-α, ASC, caspase-1, and pro-IL-1β. • Additionally, selenium hindered the activation of NLRP3 within bMECs following infection with *S. aureus.*	Yang et al. (151)
8	Selenium (1.5 mg Se/kg) treatment of *S. aureus* infected mammary gland cells	• The addition of selenium curbed the inflammation provoked by *S. aureus* within the murine mammary gland. • Moreover, selenium supplementation notably decreased the quantities of myeloperoxidase (MPO), TLR2, IL-1β, TNF-α, and IL-6 within the mammary gland of mice exposed to *S. aureus.*	Gao et al. (152)
9	Selenium (1.5 mg Se/kg) treatment of *S. aureus* infected mammary gland cells	• Selenium supplementation was effective in diminishing the quantities of NF-κB and nitric oxide, while also promoting the activation of PPAR-γ activity. • These actions collectively worked to safeguard mice from developing mastitis caused by *S. aureus* infection.	Gao et al. (136)
10	Selenium administration	• Supplementing dairy cattle with selenium substantially enhanced selenium concentrations in their serum throughout the transition phase. • Additionally, there exists an inverse correlation between Se levels and milk SCC as well as IL-6 levels. • On the other hand, Se levels are positively linked to the activity of GSH-Px in periparturient dairy cattle.	Wang D et al. (122)
11	Selenium supplementation	• Low level of selenium availability in the body was associated with higher level of SCC • Selenium administration led to a reduction in milk SCC, alleviated oxidative stress, and lowered the risk of mastitis occurrence in periparturient dairy cattle.	Ceballos-Marquez et al. (153)
12	Selenite treatment	• The introduction of selenite resulted in heightened phagocyte recruitment to the infected milk compartment of the udder, augmenting both the activity of GSH-Px and the antibacterial properties of milk lactoserum. • This supplementation also curbed the *in vitro* proliferation of mastitis-causing pathogens, indicating the potential of selenium as a potent therapeutic agent in managing mastitis.	Ali-Vehmas et al. (147)
13	Selenium feeding	• Selenium enrichment improved the effectiveness of antioxidants by fostering the activity of GSH-Px in dairy cattle from Estonia. • Additionally, observations indicated that cows receiving selenium supplementation exhibited a reduced presence of pathogenic bacteria within their milk.	Malbe et al. (148); Malbe et al. (149)

## 4 Exploring the potential of mineral nanoparticles for mastitis mitigation

In animal production, minerals can be integrated into the diet or utilized in therapy through various forms, such as inorganic salts, organic forms, chelates, or nanoparticles (NPs). Nanoparticles have been studied extensively for their potential as animal growth promoters, antimicrobials, and alternatives to conventional cleaning agents (154). A significant advantage of NPs is their ability to avoid bacterial resistance by exerting toxic effects on via DNA degradation, and lipid and protein peroxidation (155, 156). Consistently, recent studies extensively described the role of different types of nanoparticles in the treatment of mastitis with positive results (157, 158). In the literature, various types of minerals nanoparticles containing copper (CuNPs), silver (AgNPs), platinum (Pt_NPs), and zinc (ZnONPs) have been described for the treatment of mastitis (100). For instance, CuNPs have shown notable inhibitory activity against various bacteria, including *E. coli*, *Klebsiella pneumoniae*, *Pseudomonas aeruginosa, Propionibacterium acnes*, and *Salmonella typhi.* Additionally, CuNPs exhibit antifungal activity against Candida species, which can cause mastitis. Moreover, AgNPs and AuNPs have demonstrated significant susceptibility against *S. aureus* strains isolated from clinical and subclinical mastitis cases (159). Furthermore, ZnO-NPs also exhibit antimicrobial properties against *S. aureus* and other pathogenic bacteria, such as E. coli and K. pneumoniae (160). AgNPs have also been considered for use in diseases caused by algae, which are associated with udder inflammation (161). According to research by Wernicki et al. (99), AgNPs and CuNPs may have a synergistic effect on various pathogens, making them potentially effective solutions in mastitis management.

## 5 The role of vitamins supplementation in mastitis alleviation

The significance of vitamins in their capacities as antioxidants and immune regulators has been extensively deliberated in the literature (16, 150, 162). Consequently, the inclusion of vitamin supplementation in mastitis control strategies has been well-documented (163–166).

### 5.1 Role of folic acid in mastitis prevention

The existing body of published evidence strongly supports the notion that folic acid supplementation yields notable improvements in metabolic function (167), while also playing a crucial role in preventing oxidative stress and enhancing immune responses (168) in periparturient dairy cattle (169). The period around calving presents a vulnerability to lowered immunity in dairy cattle due to folic acid deficiency (165). Folic acid’s pivotal contributions to immunity and anti-inflammatory processes have led to its exploration for mitigating bovine mastitis in periparturient dairy cattle (166, 170, 171). Recently a published research demonstrated that folic acid treatment significantly regulated LnRNA MSTRG.11108.1 in mastitic cows. The LncRNA MSTRG.11108.1 further regulated the genes (CXCL3, ICAM1, CXCL1, LHFPL2, LTF, ITGA9, and KIR3DL2) that were associated with immunity and inflammation (172). In addition, they also documented several immunity and immunity linked biological signaling pathways (B cell receptor signaling pathways, TNF signaling pathway, IL-17 signaling pathway and NF-κB signaling pathway) which is in consistent with findings reported previously (165, 173). By inhibiting the activation of MAPK and NF-κB, folic acid supplementation maintains an anti-inflammatory environment, consequently averting mastitis (170). Correspondingly, a study has recorded that administering folic acid (at a dosage of 120 mg per 500 kg of body weight) for 21 days led to the downregulation of several genes linked to immune function and inflammation (PIM1, SOCS3, ATP12A, KIT, LPL NFKBIA, DUSP4, ZC3H12, ESPNL, TNFAIP3) (165). These genes, found to be upregulated in *S. aureus*-induced mastitis during the periparturient phase in dairy cattle (174, 175) were notably downregulated.

Additionally, our prior investigation established that folic acid supplementation significantly modulated the signaling of glutathione metabolism along with its associated genes (LAP3, GSR, G6PD, GSTA4, GCLC, GPX3, PGD, IDH1, GGT1, GPX7, MGST1, and MGST2) in periparturient dairy cattle (165). Moreover, we documented that folic acid effectively enhanced dairy cattle’s antioxidant capabilities and augmented their resistance to mammary gland infections during the periparturient phase. Aligning with this, (166) recently conducted an experimental study showing that *S. aureus-*induced mastitis in MAC-T cells resulted in the downregulation of the noncoding RNA associated with progenitor renewal (PRANCR). Notably, in MAC-T cells treated with folic acid, this expression exhibited an increase, suggesting folic acid’s potential as a prime therapeutic agent in mastitis prevention (166). Treatment with 5 μg/mL of folic acid significantly curtailed apoptosis in Mac-T cells and offered robust defense against MRSA treatment through enhanced cytosolic DNA sensing and tightened junction signaling (173, 176). They observed the upregulation of ZBP1, IRF3, IRF7, and IFNAR2 within the cytosolic DNA-sensing pathway in folic acid-treated MAC-T cells. ZTP1, a factor associated with milk SCC (166), also assumes a critical role in activating anti-pathogenic mechanisms and inflammation (177). Furthermore, it was found that ZTP1 gene cytosolic DNA sensing pathway upregulated by folic acid treatment which has key role in activation of antipathogenic mechanism thereby enhancing the anti-inflammatory response (173). In addition, they documented that ZTP1 was significantly associated with inhibition of inflammation (177), low milk SCC and mastitis resistance (166). In light of the compiled data, we can deduce that folic acid supplementation, administered at appropriate dosages and durations, holds promise as a valuable therapeutic resource within mastitis control strategies for periparturient dairy cattle.

### 5.2 Role of vitamin E supplementation in periparturient bovine mastitis prevention

Vitamin E is a fat-soluble vitamin that could protect cell membrane from the action of lipid peroxidation chain reaction (178, 179). Consequently, a study found that immune cells were prone to the effects of lipid peroxidation by ROS due to the polyunsaturated fatty acids present in their cell membranes (179, 180). Furthermore, the vitamin E combats peroxyl radicals and stops the oxidation of polyunsaturated fatty acids (PUFA). In the presence of vitamin E, peroxyl radicals react with α-tocopherol rather than lipid hydroperoxide, stopping the chain reaction of peroxyl radical formation and inhibiting further oxidation of PUFAs in the membrane (181). The oxidative stress caused by aluminum was relieved by post-vitamin E injection in rats (178, 182). Furthermore, it has been demonstrated that vitamin E is important for regulating immunity and reducing oxidative stress which are the key factors for mastitis resistance/susceptibility (182–185). Additionally, vitamin E has been shown to guard against pro-oxidant-caused harm to the integrity of the bovine mammary endothelial cell barrier (186). Mokhber-Dezfouli et al. demonstrated that intramuscular vitamin E injection could lower MDA expression and lipid peroxidation and enhance plasma’s antioxidant capacity. Vitamin E supplements have been given to dairy cattle during the periparturient stage to reduce oxidative stress and maintain immunity (40). In addition, vitamin E has been extensively targeted in mastitis mitigation research in periparturient dairy cows due to its substantial role as an antioxidant and immune regulator.

Vitamin E administration has been shown to improve immunity, minimize mammary infections in dairy cattle and boost the anti-inflammatory and antioxidant functions in dairy cattle during the perinatal period (189). According to Politis et al., mastitis and oxidative stress were substantially linked to vitamin E deficiency throughout the periparturient phase (190, 191). Consequently, it has been proved experimentally that supplementation of vitamin E improved the immunity, antioxidant capacity, and anti-inflammatory ability of dairy cattle, and reduced the incidences of mastitis during the transition phase in dairy cattle (192).

Parenteral injection of 2100 mg vitamin E for 14 days before and on the day of calving significantly reduced the occurrence of periparturient mastitis in dairy cattle (87). Consistently, another study reported that supplementation of 1g vitamin E/cow/day for one month pre-calving and two months post-calving reduced the incidences of mastitis in dairy cows (193). Vitamin E has also been found effective against *E. coli* and *S. uberis* and prevented mammary gland infection during the periparturient phase in dairy cows (194, 195). Altogether, it was concluded that vitamin E is the key nutrient involved in immune regulation and relieving oxidative stress, which are the factors responsible for mastitis in periparturient dairy cattle.

### 5.3 Role of vitamin D supplementation in periparturient bovine mastitis prevention

**Figure 4:**
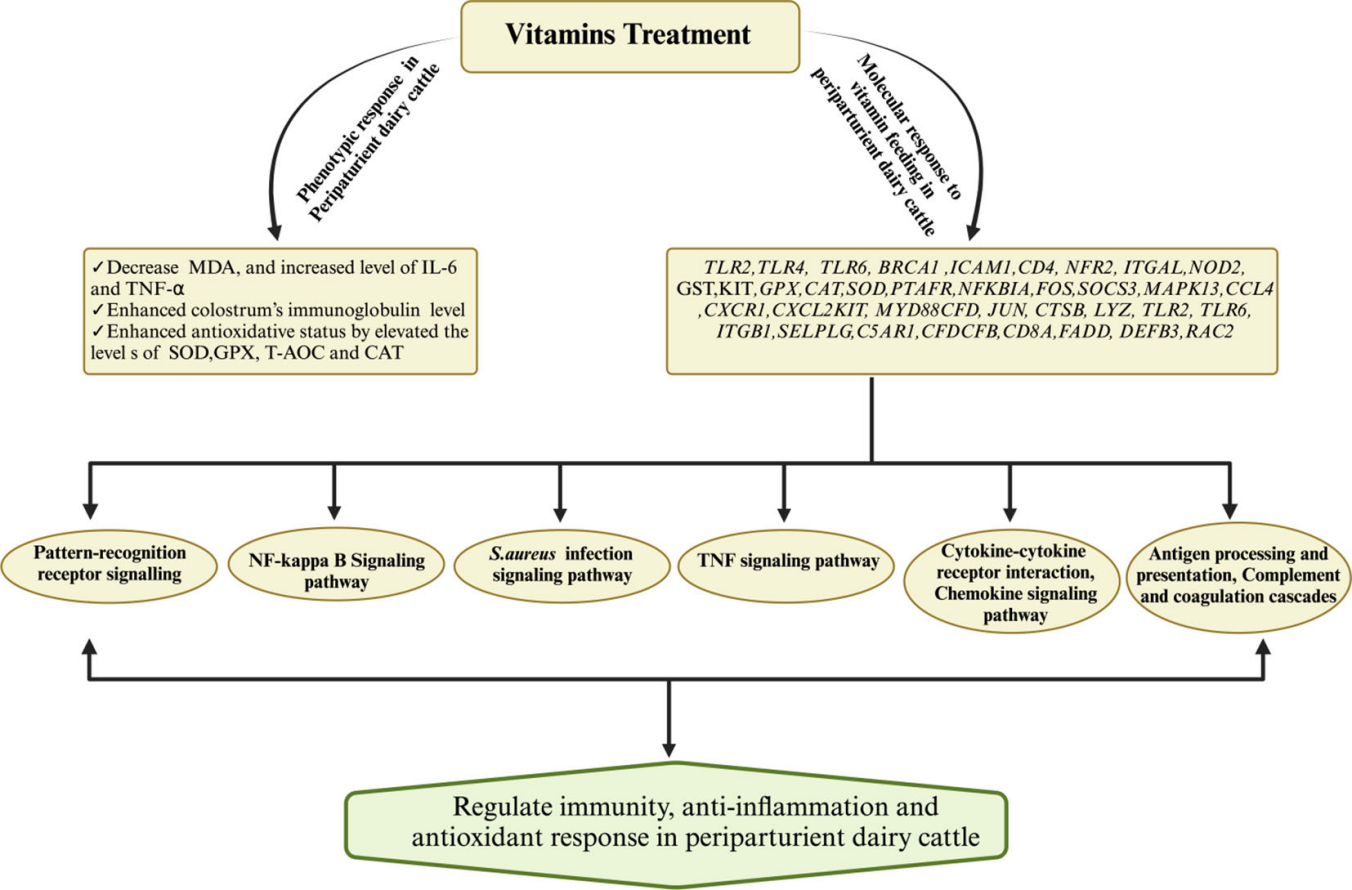
Mechanisms governing the regulation of immunity, antioxidant, and anti-inflammatory responses for the prevention of mastitis in periparturient dairy cattle through vitamin supplementation. Vitamin supplementation demonstrated an augmented antioxidative status by upregulating key antioxidant genes (SOD, CAT) while concurrently downregulating the levels of MDA. Additionally, vitamins exhibited regulatory effects on immunity-related and inflammatory-associated genes and their associated signaling pathways, thereby substantiating their pivotal role in mastitis resistance or susceptibility.

Extensive research findings have demonstrated the profound impact of vitamin D (calcidiol a source of vitamin D) administration, typically within a daily dosage range of 1mg to 3mg on the regulation of various genes associated with crucial aspects of bovine immunity, inflammation, antimicrobial activity, calcium metabolism, and oxidative response (196). Notably, genes associated with immunity (CD44, ICAM1, ITGAL, ITGB1, LGALS8, SELL, NOD2, TLR2, TLR6, FOS, JUN, NFKB2), inflammation (IL1B, IL1R1, IL1RN*)*,antimicrobial activity (CTSB, LYZ, DEFB3), calcium metabolism (TP2B1, STIM1,TRPV5, CALM3*)* and oxidative burst (RAC2)in periparturient dairy cattle were regulated in response to vitamin D supplementation (196). This regulatory effect is not limited to immune cells but extends to various tissues, as evidenced by the activation of 1α-hydroxylase and the subsequent regulation of calcitriol synthesis (197). Furthermore, exposure to bacterial components such as LPS and peptidoglycan has been observed to stimulate local expression of 1α-hydroxylase in peripheral blood monocytes of dairy cows. Additionally, the influence of vitamin D signaling has been elucidated in mammary gland macrophages and neutrophils when these cells are exposed to endotoxin challenge (198). *In vitro* experiments have also substantiated the role of vitamin D in up-regulating the mRNA activity of β-defensins and antimicrobial peptides in monocytes and milk somatic cell counts (199, 200). Importantly, studies have shown that vitamin D supplementation plays a pivotal role in reducing the incidence of mastitis and metritis in periparturient dairy cattle by mitigating oxidative stress and enhancing the immune response (201). Furthermore, vitamin D treatment has been associated with increased cell viability and the inhibition of *S. aureus* adhesion and invasion in bovine mammary epithelial cells, underscoring its potential significance in mastitis prevention (202). Consistently, a recent published article has demonstrated that vitamin D and its metabolites hydroxyvitamin D [25(OH)D] with a concentration of 20-400 ng/ml has shown a positive role on bovine immune cells, antioxidant response and could be consider as therapeutic agent for mastitis prevention (203–205).In light of the available literature, it is evident that vitamin D holds a central position in bolstering dairy cattle immunity and potentially alleviating bovine mastitis. However, it is worth noting that further comprehensive investigations are warranted to gain a deeper understanding of its precise mechanisms and potential therapeutic applications in this context. For ease of review, the recent findings associated with role of vitamins supplementation in boosting immune, antioxidant and anti-inflammatory response in dairy cattle during periparturient period has been summarized in **Table 3**. Furthermore, **Figure 4** illustrates the impact of vitamin supplementation on immune, antioxidant, and anti-inflammatory responses in dairy cattle.

**Table 3:** Role of vitamins in boosting immunity, anti-inflammation and antioxidant response in periparturient dairy cattle.

S.No	Treatment	Outcomes	References
1	Folic acid (120 mg/500 kg)/oral	Regulated immunity and suppressed inflammation via associated genes (ICAM1, GRO1 and CXCL3*)* and lncRNA MSTRG.11108.1 in periparturient dairy cattle	Liu X et al. (172)
2	Folic acid (10 µM FA/mL/cell culture)	Reduced cell apoptosis via elevation the expression of B-cell lymphoma-2 (BCL2) and the BCL2 to BCL2 associated X 4 (BAX4) in BMECs	Zhang J et al. (206)
5	Multivitamins and multiminerals (Zinc 40 mg/ml, Manganese 10 mg/ml, Copper 15 mg/ml, Selenium 5 mg/ml) and five ml of MV (Vitamin E 5 mg/ml, Vitamin A 1000 IU/ml, B-Complex 5 mg/ml, and Vitamin D_3_ 500 IU/ml)/Injection	Enhanced oxidative stress (SOD and CAT elevated), decreased anti-inflammatory cytokines (IL-4 and IL-10), increased proinflammatory cytokines (IL-1α, IL-1β, IL-6, IL-8, IL-17A, IFN-γ and TNF-α) Lower percentage of total neutrophils and immature neutrophils, higher percentage of lymphocytes as well as increased phagocytic activity of neutrophils and proliferative capacity of lymphocytes	Somagond et al. (44)
3	Folic acid (30.8 ng/mL/cell culture)	Reduced apoptosis by enhancing bcl-2/bax mRNA expression	Bae et al. (176)
4	Vitamin E and folic acid	Regulate immunity, antioxidative stress and relieved inflammatory responses and prevent mastitis	Khan et al. (16) and Xiao et al. (36)
6	Folic acid (5 μg/mL/cell culture)	Mediated the expression alteration in lncRNAs linked to toxin metabolism and inflammation to fight against *S. aureus* infection	Mi et al. (166)
7	Folic acid (120 mg/500 kg)/oral	Relieved oxidative stress, enhanced immunity and anti-inflammatory status, reduced SCC level and improved milk production	165
8	Folic acid alone or in combination with vitamin B12/(120 mg/500 kg)/oral	Enhanced the perimarturient dairy cattle health and production performance (enhanced immunity and milk production)	167, 169
9	Calcidiol 3 mg/d (3mg) prepartum	Regulated bovine immunity, anti-inflammatory response, antimicrobial activity leukocytes, pathogen recognition efficacy, calcium metabolism, and antioxidative status of periparturient dairy cattle	Vieira-Neto et al. (196)
10	25-hydroxyvitamin D3 (3 mg/oral)	Lowered the level of SCC and enhanced T-AOC, total SOD, CAT, immunoglobulin A and immunoglobulin G and decreased MDA and TNF-α. Reduced the susceptibility of periparturient dairy to mastitis	Xu et al. (207)
11	Dietary protein levels (10.3% or 12.2%), vitamin A levels (0 or 110 IU/kg body weight),	Lowered the level of SCC and improved ant-inflammatory response in periparturient dairy cattle	Agustinho et al. (69)
12	The supplementation of vitamin A, 10^5^ IU + zinc sulphate, 60 ppm+ vitamin E, 2500 IU) in compounded concentrate DM (100 g)/oral	Lowered milk SCC and enhanced total immunoglobulins in colostrum Lowered oxidative stress and cortisol levels and higher immune response Improved the phagocytic activity of neutrophils of periparturient dairy cattle	Alhussien et al. (70)

## 5 The effect of rumen-protected amino acids on immune function, oxidative and anti-inflammatory status of dairy cattle

The cellular detoxification process is facilitated by GSH through the action of GST and the neutralization of hydrogen peroxide by GSH-Px. The GSTs (EC 2.5.1.18) are pivotal antioxidant enzymes responsible for the regulation of cellular redox equilibrium, as documented by several studies (208–210). Recent evidence underscores the involvement of methionine in glutathione synthesis (211), thereby potentially enhancing the antioxidant capacity in animals and their products. This notion is corroborated by research demonstrating that methionine, in conjunction with choline, elevates glutathione and amino acid levels in perinatal dairy cows (212). Furthermore, methionine supplementation has been found to augment very-low-density lipoprotein (VLDL), facilitating the circulation of vitamin E (213). Consequently, the detrimental impact of lipid peroxidation by-products, such as MDA, can be mitigated through the administration of rumen-protected amino acids (213). Additionally, the control of ROS by antioxidant systems, categorized into enzymatic and non-enzymatic components like metabolites, has been extensively discussed in previous studies (210, 214).

Dysregulated immune function, notably in early lactation dairy cows, has been observed, adversely affecting neutrophils, circulating monocytes, and lymphocytes (6, 215 55, 216). It is postulated that complete metabolic adaptations are required to cope with the substantial nutrient demands associated with lactation initiation, contributing to immunological dysfunction (19, 217, 218). A noteworthy finding is that prolactin blockade leads to an increase in oxidative burst activity in neutrophils, resulting in an initial reduction in milk production and nutritional requirements, along with a subsequent decline in lymphocyte proliferation (219).

Empirical investigations have consistently demonstrated that methionine supplementation exerts favorable effects on the anti-inflammatory and antioxidant status in periparturient dairy cattle (220–222) and neonatal calves (223). A recent study conducted by Hu et al. has reported that the supply of methionine and arginine significantly modulates milk protein synthesis, thereby alleviating potential inflammatory and pro-oxidant conditions in transition dairy cattle (224). Moreover, emerging research has highlighted the significance of methionine supplementation in conjunction with arginine in conferring anti-inflammatory effects and enhancing the antioxidant status in transition dairy cattle. Notably, Dai et al. (225) and Batistel et al. (222) have reported the beneficial impact of methionine and arginine co-supplementation. Additionally, Abdelmegeid et al. (226) documented that the combination of choline and methionine effectively regulates antioxidative mechanisms, resulting in heightened anti-inflammatory and cytoprotective responses against oxidative stress in neonatal Holstein calves. Consistently, Zhou et al. (227) demonstrated that choline and methionine supplementation improved immunometabolic status, bolstered blood polymorphonuclear leukocyte phagocytosis capacity, promoted anti-inflammatory responses upon pathogenic challenges, and elevated antioxidative capacities in peripartal cows. In a relevant experiment, a group of cows was subjected to either methionine supplementation alone or in combination with lysine. Wang H et al. (223) found that the offspring (calves) of cows receiving rumen-protected amino acids exhibited heightened passive immunity, characterized by increased immunoglobulin G concentrations and superior growth rates compared to their counterparts in the unsupplemented amino acid group. Furthermore, they documented that methionine supplementation upregulated the expression of antioxidant-related genes, such as SOD and GSH-Px and conferred cytoprotective effects against hyperthermia (228, 229). The synergistic effects of methionine and arginine supplementation have also been observed in the regulation of immunity and the mitigation of oxidative stress induced by bacterial LPS in BMECs (225). Dai et al. (225) reported that bacterial LPS significantly down-regulated the expression of key genes associated with antioxidant responses, such as NFE2L2, NQO1, GPX1, ATG7, and GPX3, while increasing the levels of SOD2 and NOS2 in BMECs. Folic acid was found to enhance antioxidant activity, reduce the expression of inflammation-related genes, and improve udder health in dairy cattle (225). Additionally, when BMECs were challenged with gamma-d-glutamyl-meso-diaminopimelic acid (iE-DAP), a component of bacterial cell walls, it induced inflammatory changes and oxidative stress, which were effectively mitigated by arginine and methionine treatment (230). Similarly, another study demonstrated that glutamine treatment provided protective effects against the adverse effects of iE-DAP in BMECs (231). Furthermore, iE-DAP was found to elevate the expression of inflammatory markers, including NOD1, inhibitor of nuclear factor-κB (NFKBIA, IκB), nuclear factor-κB subunit p65 (RELA, NF-κB p65), IL-6, and interleukin-8 (IL-8) in cell culture (231). However, when cells treated with iE-DAP were subsequently exposed to glutamine, this intervention led to the suppression of the NOD1/NF-κB pathway and the enhancement of antioxidant protein levels (231). This aligns with previous findings that amino acids play a pivotal role in NO regulation in mammary cells, thereby exerting antibacterial activity against LPS during inflammation (232–234). *In vitro* experiments have consistently demonstrated that methionine supplementation effectively attenuates apoptosis, necrosis, and lipid peroxidation in bovine mammary gland cells (228, 229, 235). Moreover, these studies have shown that arginine and methionine co-supplementation enhances the expression of antioxidative genes and elevates the NFE2L2 signaling pathway in mammary cells, a crucial component of the cellular antioxidant defense system (236). Lan et al. (237) conducted experimental research revealing that pretreatment with 2 mM Met-Met had the capacity to mitigate the elevated levels of specific inflammatory markers following exposure to 1 μg/mL LPS. This included a reduction in TNF-α, IL-1β, and IL-8. Furthermore, their investigation indicated that the genes commonly affected by this treatment were primarily associated with the NF-κB, MAPK, and IL-17 pathways. Notably, the suppression of NF-κB, P38, and JNK by Met-Met appeared to occur through the Janus kinase 2-signal transducers and activators of transcription 5 (JAK2-STAT5) pathway (237). Additionally, it was observed that the Met-Met-induced reduction in LPS-triggered activation of p-IκB, NF-κB, and JNK was reversed in the presence of a JAK2 inhibitor, highlighting the intricate interplay between Met-Met and these signaling pathways (237). Furthermore, a concise summary of research endeavors investigating the impact of rumen-protected amino acids on immune function, oxidative stress, and anti-inflammatory responses in dairy cattle has been thoughtfully compiled in **Table 4** for your reference and elucidation.

**Table 4:** Summary of studies investigating the effect of rumen-protected amino acids supplementation on immune function, oxidative and anti-inflammatory status in dairy cattle.

S.No	Amino acid Administration	Main outcomes	References
1	Methionine and lysine supplementation (107g/twice a day/7 weeks/oral)	• Attenuated the severity of SCC and BCS, thereby mitigating the risk of clinical mastitis occurrence.	Abreu et al. (238)
2	Methionine/at a rate of 0.09% and 0.10% of DMI/oral/28 day before parturition till 60 days after parturition (88 days)	• Notable enhancement in plasma biomarkers, following improved liver function, reduced oxidative stress and inflammation, as well as enhanced oxidative burst and neutrophil phagocytosis.	Batistel et al. (222)
3	Increase methionine (175 μg/mL) and lysine (175 μg/mL) ratio 1:2.5 respectively	• Modulated the expression of genes associated with immunity, anti-inflammation, and antioxidation in LPS-challenged BMECs. • Elevated the expression of genes such as NFE2L2, NQO1, GPX1, GPX3, SLC36A1, SLC7A1, SOD2, NOS2, and concurrent decreased expression of RELA, IL1B, NF-kb, and CXCL2.	Dai et al. (225)
4	5-10 gm zinc methionine/head/day/oral	• Reduced the SCC levels and decreased the required mastitis treatment duration with antibiotics	Gaafar et al. (239)
5	Methionine at a rate of 0.09% and 0.10% of DMI/oral/28 day before parturition till 60 days after parturition (88 days)	• Upregulated the expression of genes involved in the metabolism of antioxidants, notably increasing the expression level of NFE2L2, a prominent transcription factor associated with antioxidant response.	Han et al. (240)
6	Methionine at a rate of/0.08% of DM/d/oral	• Reduced inflammatory changes, improved antioxidant status followed by augmentation of immune responses, thereby reducing the susceptibility to infections in transition neonatal calves	Jacometo et al. (241); Jacometo et al. (242) & Jacometo et al. (243)
7	2 mM methionyl-methionine treatment	• Mitigated the inflammatory alterations induced by LPS through downregulation of the expression of inflammatory-associated genes, including *TNF-α, AP-1, MCP-1, Jak2, IL-1β*, and *IL-8*, along with the inhibition of key signaling pathways such as *JAK2-STAT5, NF-κB, MAPK*, and *IL-17* in BMECs	Lan et al. (237)
8	2 mM methionyl-methionine treatment	• Significantly reduced the expression of inflammatory linked genes such as *IL-8*, *TNF-α*, *AP-1*, and *MCP-1* induced by LPS in BMECs	Lan et al. (244)
9	Rumen-protected lysine (10 g of digestible lysine/cow per day) and methionine (4 g of digestible Methionine/cow)	• Reduced the levels of BHB, improved BCS, and lowered SCC, which collectively contributing to enhanced udder health in periparturient dairy cattle	Lee et al. (245)
10	Hydroxyselenomethionine supplementation (0.5 mg/oral)	• Significantly elevated the levels of antioxidative status and reduced the risk of periparturient mastitis in cattle	Li et al. (246)
11	Methionine supplementation at a rate of 0.09%/oral	• Enhanced the mRNA expression of genes associated with antioxidative status and the metabolism of GSH, thereby improving cellular antioxidant defenses.	Liang et al. (247)
12	60 g/d of NALM acetyl-l-methionine (NALM)/cow	• Ameliorated oxidative stress in mid-lactating dairy cows, as evidenced by increased concentrations of total plasma protein and globulin, concomitant with a reduction in plasma MDA concentration.	Liang et al. (248)
13	Methionine and choline treatment/cell culture	• Significantly enhanced the expression of genes linked to immunity and anti-inflammatory responses, while concurrently reduced oxidative stress in bovine PMNLs	Lopreiato et al. (249)
14	Methionine supplementation at the rate of 0.19 or 0.07% DMI	• Enhanced whole blood neutrophil phagocytosis followed by improved immune defense system • Reduced oxidative stress and improved antioxidant response in dairy catle • The improved immunity and antioxidant status were associated with reduced risk of mastitis	Osorio et al. (250)
15	Methionine supplementation at the rate of 0.19 or 0.07% DMI	• Triggered a series of positive metabolic changes with a notable enhancement of 1-carbon metabolism, showcasing its impact on fundamental metabolic processes. • Antioxidant status was improved, as evidenced by elevated levels of liver GSH. • Decreased concentrations of plasma biomarkers associated with inflammation, indicating its potential role in mitigating inflammatory responses and promoting overall physiological well-being	Osorio et al. (211); Osorio et al. (220)
16	Zn-Meth supplementation (1 g/d Zn/oral)	• **Reduced inflammatory changes, enhanced immunity and lowered the SCC following improved** udder health in transition dairy goats	Salama et al.
17	Increase methionine (175 μg/mL) and lysine (175 μg/mL) ratio 1:2.5 respectively	• Regulated the **AKT1 and mTORC1 Signaling** which are pivotal in cell growth, proliferation, and immune responses. • In addition elevated the expression of genes associated with anti-inflammatory responses and metabolic regulation (***PPARG***), anti-apoptotic activity (CL2L1) and cell growth and immunity (***MAPK1 MTOR, RPS6KB1, BAX, EIF4EBP1* and *JAK2***)	Salama et al. (252)
18	Methionine (30 g/d of Mepron)/oral supplementation	• Improved immune system’s cellular defense mechanisms by enhancing population of T lymphocytes in the bloodstream. • Reduced the level of SCC, which shows methionine supplementation could significantly prevented mastitis	Soder and Holden, (253)
19	15 g/d RPC + 15 g/d RPM from 21 days prepartum to 21 days postpartum.	• Enhanced CD4+/CD8+ T, GSH-Px, T-AOC, decreased the plasma concentrations of NEFA, BHBA, total cholesterol (TC) and low-density lipoprotein cholesterol (LDL-C) • In addition, lymphocyte ratio, immunity and antioxidative status were improved	Sun et al. (213)
20	Methionine (8.0 and 12.0 g/d) and choline (12.4 g/d) supplementation	• Effectively mitigated the hyperactive response of IL-1β during an LPS challenge. This suggests that methionine improved the body’s ability to regulate inflammatory processes, potentially reducing the risk of excessive inflammation and its associated negative effects. • Relieved oxidative stress and improved postpartum neutrophil and monocyte phagocytosis capacity. • In addition phagocytosis and oxidative burst activity was improved. This means that immune cells were more effective in generating reactive oxygen species to destroy pathogens, further strengthening the immune response.	Vailati-Riboni et al. (254)
20	Infusion of arginine (3gm/h for 5 days) protected the dairy cattle from LPS challenge (0.033 μg/kg per h)	• Relieved oxidative stress and inflammatory changes caused by LPS in dairy cattle. Arginine infusion played a role in mitigating inflammation triggered by LPS. • Suppressed the LPS induced NOS and reduced the LPS-binding protein levels. This is significant because LPS-binding protein is associated with the body’s recognition of bacterial endotoxins, and reducing its levels may contribute to a reduced risk of systemic inflammation.	Zhao et al. (255)
21	Infusion of arginine (3gm/h for 5 days) protected the dairy cattle from LPS challenge (0.033 μg/kg per h)	• Enhanced the body’s antioxidant defenses. This could be due to its properties as a precursor to NO, which has antioxidant effects. • Enhanced the level of TAC which suggests that arginine contributes to the body’s ability to counteract oxidative stress during inflammation. • Increased the expression of GSH and inhibited the MDA level in LPS challenged dairy cattle	Zhao et al. (256)
22	Methionine and choline supplementation (at a rate of 0.08% of DM/oral)	• Regulates taurine synthesis, which is involved in supporting antioxidant defenses and stabilizing cell membranes. • Being a precursor for GSH, play a key role in enhancing antioxidant status. • Reduced inflammatory and chances of udder infections in periparturient dairy cattle	Zhou Z et al. (221)
23	Methionine supplementation at 0.08% of DMI/oral l	• Improved biochemical pathways, including DNA methylation, which can influence gene expression. Increased methionine availability can potentially affect the expression of genes related to different biological processes (immunity, inflammation and oxidative stress) • Strengthening the immune system by upregulating the expression of genes associated with immunity. • Enhanced the body’s antioxidant defenses by influencing the expression of antioxidant linked.	Zhou et al. (212)
24	Methionine supplementation at 0.08% of DMI/oral	• Significantly decreased the expression of genes associated with inflammation and oxidative processes and improved the udder health in periparturient dairy cattle	Zhou et al. (227)

## 6 Future research direction

While this review has synthesized existing knowledge regarding the role of amino acids, vitamins, and trace minerals in mitigating periparturient mastitis, several avenues for future research emerge. Further investigation into the intricate mechanisms underlying the interactions between these nutrients, oxidative stress, and immune modulation will deepen our understanding of their synergistic effects. Incorporating molecular and cellular approaches can unravel the specific pathways through which these nutrients influence immune responses and oxidative balance. Additionally, longitudinal studies assessing the long-term effects of targeted supplementation on mastitis incidence, milk quality, and overall cow health are essential to validate the efficacy of these strategies under practical farming conditions. Furthermore, understanding the interplay between genetic factors, environmental conditions, and nutritional interventions can provide insights into personalized approaches for mastitis prevention. Moreover, exploring novel delivery mechanisms for these nutrients, such as innovative formulations or precision feeding, could optimize their absorption and utilization within the complex physiological milieu of periparturient dairy cattle. In conclusion, future research endeavors should focus on unraveling the nuances of nutrient-nutrient interactions, delineating precise molecular mechanisms, and translating these findings into practical strategies that empower dairy farmers to effectively manage periparturient mastitis, bolstering animal health and farm productivity.

## 7 Conclusion

In light of the intricate interplay between immune suppression, oxidative stress, and metabolic perturbations during the periparturient period, this comprehensive review underscores the paramount importance of proactive nutritional strategies in mitigating bovine mastitis. The critical vulnerabilities arising from negative energy balance, oxidative stress, and compromised immune responses underscore the need for targeted interventions to enhance udder health and overall productivity in dairy cattle. The exploration of trace minerals, vitamins, and amino acids as key mitigation factors offers promising avenues for addressing periparturient bovine mastitis. These nutritional components have demonstrated significant potential in bolstering antioxidant defenses, modulating immune responses, and preventing oxidative damage. The multifaceted roles of trace minerals, particularly copper, selenium, and calcium, have been deliberated in the context of mastitis control. Similarly, the established impact of vitamins, such as vitamin B12 and vitamin E, in enhancing metabolic function and immune responses underscores their potential in ameliorating mastitis susceptibility. Furthermore, amino acids’ pivotal role in maintaining cellular oxidative balance through their participation in crucial biosynthesis pathways presents a novel perspective in combatting mastitis-related challenges. The insights gained from this review highlight the need for a holistic approach that encompasses these nutritional factors to enhance udder health and mitigate the risks associated with periparturient mastitis.

## Author contributions

MK: Conceptualization, Data curation, Formal analysis, Methodology, Supervision, Validation, Visualization, Writing – original draft, Writing – review & editing. BH: Conceptualization, Data curation, Writing – original draft, Writing – review & editing. XK: Data curation, Writing – review & editing. YC: Data curation, Writing – review & editing. HL: Data curation, Writing – review & editing. QU: Data curation, Writing – review & editing. IK: Data curation, Writing – review & editing. AK: Data curation, Writing – review & editing. WC: Data curation, Writing – review & editing. CW: Conceptualization, Data curation, Funding acquisition, Project administration, Resources, Supervision, Validation, Visualization, Writing – original draft, Writing – review & editing.
